# Radiotherapy is Important for Local Control at Primary and Metastatic Sites in Pediatric Rhabdomyosarcoma

**DOI:** 10.7759/cureus.388

**Published:** 2015-11-23

**Authors:** Sonia Skamene, Sharon Abish, David Mitchell, Carolyn Freeman

**Affiliations:** 1 Department of Radiation Oncology, McGill University Health Centre; 2 Department of Pediatric Hematology-Oncology, McGill University Health Centre

**Keywords:** radiation oncology, pediatric tumors, soft tissue sarcoma, rhabdomyosarcoma

## Abstract

Purpose: The current recommended practice for pediatric patients with metastatic rhabdomyosarcoma includes full-dose radiotherapy to each metastatic site. We wished to question this practice, which can cause side-effects and is often logistically challenging, by studying the pattern of failure in our pediatric and teenage patient population.

Methods and Materials: Our institution’s cancer registry was queried for patients diagnosed with rhabdomyosarcoma aged 18 or less from January 1990 until January 2014. Twenty-nine patients were found and, of these, six had metastatic disease. Five of the six were treated with standard chemotherapy together with radiotherapy to the primary and metastatic sites with doses and fractionation according to the site. Progression-free survival was calculated from the end of radiotherapy until radiological or pathological evidence of disease progression or death.

Results: Median age was 13 years (range: 12-18). Three were girls. All had alveolar histology and unfavorable primary sites. Twelve metastatic sites were treated with radiotherapy. Doses used were 41.4 - 50.4 Gy in 1.8 Gy fractions for most sites, and 15 Gy in 1.5 Gy fractions for whole lung radiotherapy. The median number of sites treated per patient was two (range: 1 - 6). Median time to progression was 10.1 months (range: 1.9 - 15.7). Local control was 100% for all metastatic sites. Median overall survival (OS) was 31.8 months (range: 20.4 – 95.4 months). Three patients developed progressive disease outside the treated field. One patient died from a secondary hematological malignancy without evidence of disease progression. One patient remains progression-free at 88.6 months post-radiotherapy.

Conclusions: Radiotherapy to metastatic disease sites prevented in-field progression in all five patients with metastatic alveolar rhabdomyosarcoma. However, failure at sites outside of the radiotherapy volume occurred in three of five of patients and overall survival was very poor despite aggressive treatment to all sites of disease. Radiotherapy has a role in metastatic disease, although future studies evaluating dose and fractionation are needed.

## Introduction

Rhabdomyosarcoma (RMS) is the most common soft-tissue sarcoma in the pediatric and adolescent age group. Although survival for patients with limited stage RMS has improved significantly over the past 20-30 years, the same is not true for the 15% of patients who present with metastatic disease [1]. Children with limited stage disease have a five-year overall survival (OS) of 70% compared to 30% for those with metastases [1-2]. 

Treatment for patients presenting with metastases consists of intensified chemotherapy, together with radiotherapy (RT) or surgery to the primary site and radical dose of RT to all metastatic sites. This has been the standard in the Intergroup Rhabdomyosarcoma Study Group (IRSG) studies since 1972. While some of these studies have explored the timing of RT, no study – IRSG or other – has challenged the need for a curative dose of RT to all metastatic sites. Given the fact that such treatment may cause significant acute side-effects as well as the logistical challenges, we analyzed our experience with control at metastatic sites after such treatment. 

## Materials and methods

After ethics approval from the McGill University Health Centre Institutional Review Board, we queried our institution’s cancer registry for patients aged 0-18 years diagnosed with metastatic RMS from January 1990 until January 2014. Informed consent was waived for this study. Local control (LC) was assessed retrospectively on follow-up imaging studies, including computed tomography (CT) scan, magnetic resonance imaging (MRI), and fluorodeoxyglucose-PET (FDG-PET). Imaging was performed to assess the post-treatment response and control at metastatic sites. Progression-free survival was calculated from the end of radiotherapy until radiological or pathological evidence of disease progression or death. Overall survival was calculated using the Kaplan-Meier method.

## Results

Six patients with metastatic RMS were identified. One child was excluded as he received only palliative RT. Patient characteristics and treatment details are given in Table 1. Median age at diagnosis was 13.8 years (range: 12.1 - 18.0 years). Primary tumor location was unfavorable for all (extremities, nasopharynx, and chest wall). The pathology for all patients was alveolar RMS. There were 14 metastatic sites at presentation, with a median of two sites per patient (range: 1 - 6 sites). No patient had bone marrow involvement at diagnosis.

**Table 1:** Characteristics, treatment details, and outcomes for the five patients included in the analysis

Case	Sex	Age	Primary Site	Metastatic Sites	Metastatic Sites and Doses (Total Dose (Dose Per Rraction))	Failure Sites	Time to Progression (Months)
1	M	18	Hand	1. T9 vertebra 2. Axillary lymph nodes (LN)	1. Axilla: 50.4 Gy (1.8) 2. T9: 45 Gy (1.8)	Liver Para-aortic LN Calvarium Sternum	15.5
2	F	13	Foot	1. Popliteal LN 2. Inguinal LN 3. Iliac LN 4. Paraaortic LN 5. Breast (surgically resected) 6. Bone (axial skeleton)	1. Popliteal LN: 41.4 Gy (1.8) 2. Inguinal LN: 36 Gy (1.8) 3. Iliac LN: 36 Gy (1.8) 4. Paraaortic LN: 36 Gy (1.8)	Abdominal wall Breast Brain Spine	2
3	M	12	Naso-pharynx	1. Lungs	1. Whole lung – 15 Gy (1.5)	none	n/a (died from AML, no evidence of RMS at death 14 months post-RT)
4	F	16	Hand	1. Axillary LN 2. Internal mammary LN	1. Axillary LN: 45 Gy (1.8) 2. IMC: 45 Gy (1.8)	none	n/a (no evidence of disease after 88 months)
5	F	13	Chest wall	1. Mediastinal LN 2. Lungs 3. Breast	1. Mediastinal LN: 41.4 Gy (1.8) 2. Whole lung: 15 Gy (1.5)	Flank (contralateral) Extensive abdomino-peritoneal disease	6

All patients were treated on or as per the recent Children's Oncology Group (COG) protocol and received multiagent chemotherapy. Three received vincristine, doxorubicin, and cyclophosphamide with etoposide and ifosfamide. Two received vincristine, actinomycin D, and cyclophosphamide, one with and one without irinotecan. The timing of local treatment and of metastatic sites was as per protocol. Four patients had definitive RT to the primary site and one had RT after surgery. Two metastatic sites in the breast were surgically resected. The remaining 12 sites treated with RT included lymph node regions (7), bone (3), and lung (2). RT was given to each site irrespective of chemotherapy response, although treatment volume and dose were adjusted according to residual disease. No patients progressed on chemotherapy prior to receiving RT. Two patients received whole-lung RT to 15 Gy. One had no residual pulmonary disease post-chemotherapy, and the other had a significant response with only a few millimetric lesions remaining. The median dose of radiotherapy to other sites of metastatic disease was 41.4 Gy (range: 36 to 50.4 Gy).

Median OS was 31.8 months (range: 20.4 – 95.4 months). One patient died from treatment-related acute myeloid leukemia (AML) at 20.4 months without recurrence of RMS. A second patient also developed AML. She underwent stem cell transplantation and shortly thereafter, at 37 months post-diagnosis of RMS, developed biopsy-proven recurrent RMS at other sites. One patient is alive without evidence of disease after more than seven years. One patient failed at a new site just outside the treated volume that consisted of bilateral lung fields treated to 15 Gy with a boost to a right chest wall tumor bed and an epicardiophrenic mass to 45 Gy. The region of failure in the left flank (contralateral to the primary tumor) had received a median dose of 5.7 Gy (range: 0.8 - 9.6 Gy). Median progression-free survival was 10.1 months (range: 1.9 – 15.7 months). Three patients had disease progression during the follow-up interval and died from the disease. There were no in-field recurrences (100% LC).

Radiotherapy was well-tolerated with minimal acute side-effects, other than mild to moderate fatigue, in all five patients. One patient experienced Grade IV skin toxicity at the primary site (the hand). She recovered well and has had a good cosmetic and functional outcome in long-term follow-up. The remaining patients had only Grade I or II skin toxicity.

## Discussion

There has been no significant improvement in outcome for patients with metastatic RMS since the beginning of multi-institution trials in North America and Europe despite intensified chemotherapy and curative RT to metastatic sites. Based on available evidence, it remains unclear whether aggressive treatment of metastatic sites is indeed beneficial to patients in terms of survival and quality of life.

Aggressive local treatment to the primary tumor was shown in a retrospective analysis to improve RMS outcomes, particularly with the use of multimodality therapy [3]. Data from studies on Ewing’s sarcoma (ES) similarly demonstrate the importance of LC to the primary site [4-5]. Although the use of whole lung radiotherapy is widely accepted due to its benefits in patients with ES pulmonary metastases as well as RMS [6-7], few studies have specifically examined LC at metastatic sites in RMS. In one series of 13 patients with metastatic ES or RMS, LC for extrapulmonary metastases was 92% at five years, with only one failure during the follow-up period [8]. Casey and colleagues retrospectively evaluated dose and efficacy for RT to bone metastases in patients with RMS and ES [9]. They found 92% LC at metastatic sites, with one failure out of 13 treated bony metastases amongst patients with RMS. They used a variation of doses and fractionation regimes and determined that neither biologically equivalent dose (BED) nor fractionation had an impact on LC. Their three-year event-free survival and OS rates for patients with RMS were 33% and 45%, respectively. These findings are similar for patients with metastatic ES. Hausler and colleagues, for example, report event-free survival of 36% at three years for patients who received RT to local and metastatic sites versus 16% for those who did not. LC at metastatic sites in this study was not evaluated [4]. There are no studies looking at the role of RT for metastatic sites in only patients with RMS.

This small series demonstrated excellent LC at both primary and metastatic sites. LC at the primary site was better than in the third Intergroup Rhabdomyosarcoma Study (IRS-III) [10] and fourth Intergroup Rhabdomyosarcoma Study (IRS-IV) [11], two large North American cooperative group clinical trials, which reported 19% and 13% local failure, respectively, and LC at metastatic sites exceeding 90%. Of note, LC at metastatic sites was achieved using doses equal to and lower than 45 Gy for nine of the 10 extrapulmonary disease sites in our experience. In some cases, we guided our dose selection using chemotherapy response on pre-radiotherapy FDG-PET, which has been shown to predict outcomes in RMS [12-13].

Our results are limited by the small number of patients included in this series. Due to the poor prognosis of these patients and short observation interval after treatment, we were unable to evaluate long-term control of radiotherapy to metastatic disease sites for four of the five patients.

Nonetheless, it is evident that RT was effective in controlling local disease and aggressive management of metastatic disease seems appropriate. However, the optimal dose and fractionation have not been defined, and whether the local control benefit extends to an overall survival benefit remains to be seen. There would be significant advantage to reducing the total RT dose and number of fractions/treatment duration to metastatic sites in patients with RMS since, when treating multiple sites, the duration of daily treatment and the four to six week overall treatment time is taxing on the patient and his/her family as well as on the RT department. Additionally, the risk of acute and long-term RT complications, including functional outcomes and secondary malignancies, increases with increasing dose and may be more relevant with patients with limited metastatic disease and excellent response to treatment. Dose de-escalation based on response to chemotherapy and/or hypofractionation could be considered in future trials with the goal of maintaining a high LC rate with the goal of prolonging survival while hopefully improving the quality of life. 

## Conclusions

Radiotherapy to metastatic disease sites prevented in-field progression at 12 metastatic sites in five patients with metastatic alveolar rhabdomyosarcoma. In spite of this high rate of local control, three patients had failure outside the treated fields and patient survival was very poor. Radiotherapy is effective at locally controlling metastatic sites in this disease, but the optimal dose and fractionation needs to be better defined.
